# A positive feedback regulatory loop, SA-*AtNAP*-*SAG202/SARD1*-*ICS1*-SA, in SA biosynthesis involved in leaf senescence but not defense response

**DOI:** 10.1186/s43897-022-00036-x

**Published:** 2022-06-17

**Authors:** Yaxin Wang, Bin Liu, Youzhen Hu, Su-Sheng Gan

**Affiliations:** 1 Sections of Plant Biology, School of Integrative Plant Science, Cornell University, Ithaca, New York, 14853 USA; 2 Present address: Nobell Foods, South San Francisco, California, 94080 USA; 3 Present address: College of Food Science, Shihezi University, Xinjiang, 832000 China

**Keywords:** Aging, Biotic stress, Feedback regulation, Leaf longevity, Salicylic acid (SA), Senescence-associated gene (*SAG*)

## Abstract

Salicylic acid (SA) is an important plant hormone that regulates defense responses and leaf senescence. It is imperative to understand upstream factors that regulate genes of SA biosynthesis. SAG202/SARD1 is a key regulator for *isochorismate synthase 1* (*ICS1*) induction and SA biosynthesis in defense responses. The regulatory mechanism of SA biosynthesis during leaf senescence is not well understood. Here we show that AtNAP, a senescence-specific NAC family transcription factor, directly regulates a senescence-associated gene named *SAG202* as revealed in yeast one-hybrid and *in planta* assays. Inducible overexpreesion of *AtNAP* and *SAG202* lead to high levels of SA and precocious senescence in leaves. Individual knockout mutants of *sag202* and *ics1* have markedly reduced SA levels and display a significantly delayed leaf senescence phenotype. Furthermore, SA positively feedback regulates *AtNAP* and *SAG202*. Our research has uncovered a unique positive feedback regulatory loop, SA-*AtNAP*-*SAG202*-*ICS1*-SA, that operates to control SA biosynthesis associated with leaf senescence but not defense response.

**Supplementary Information:**

The online version contains supplementary material available at 10.1186/s43897-022-00036-x.

## Core

A unique positive feedback regulatory loop, SA-*AtNAP*-*SAG202/SARD1*-*ICS1*-SA is found to operate and modulate SA biosynthesis to regulate leaf senescence in Arabidopsis. Although part of the loop, *SAG202/SARD1*-*ICS1*-SA is shared by defense response, the whole loop is not responsive to pathogen attack.

### Gene & accession numbers

Sequence data from this article can be found in the GenBank/EMBL databases under the following accession numbers: AT1G73805 (*SAG202*, *SARD1*), AT4G10500 (*S3H*), AT1G69490 (*AtNAP*), AT1G74710 (*ICS1*), AT1G18870 (*ICS2*), AT5G26920 (*CBP60g*) and AT3G18780 (*Actin2*, *ACT2*).

## Introduction

Salicylic acid (SA, 2-hydroxy benzoic acid) has pivotal roles in the regulation of many aspects of plant growth and physiological processes such as defense responses, thermogenesis, seed germination, flowering and senescence (Raskin, 1992; Rivas-San Vicente and Plasencia, 2011). It is generally accepted that there are two SA biosynthesis pathways in plants: the isochorismate (IC) pathway and the phenylalanine ammonia-lyase (PAL) pathway (Métraux, 2002; Chen et al., 2009b). In Arabidopsis, the IC pathway contributes to most of the SA production induced by pathogens and UV light (Garcion et al., 2008; Dempsey et al., 2011). Although two genes, namely *isochorismate synthase 1* (*ICS1*) and *ICS2,* are involved in isochorismate synthesis in the IC pathway, *ICS1* accounts for approximately 90% of the total amount of isochorismate produced in response to pathogens or UV light (Surplus et al., 1998; Wildermuth et al., 2001; Garcion et al., 2008). It is known that SA levels increase with progression of leaf senescence (Morris et al., 2000; Zhang et al., 2013; Zhang et al., 2017b); however, whether the IC pathway operates and functions during leaf senescence is not well known.

The regulation of SA biosynthesis and the SA signaling in local and systemic acquired resistance (LAR and SAR) responses against pathogens have been intensively investigated (Shirasu et al., 1997; Dangl, 1998; Shah, 2003). Ethylene insensitive 3 (EIN3) and EIN3-like 1 (EIL1) suppress *ICS1* to negatively regulate SA biosynthesis (Chen et al., 2009a), and two closely related transcription factors, calmodulin binding protein 60 g (CBP60g) and systemic acquired resistance deficient 1 (SARD1), bind to the core sequence 5’GAAATTTTGG3′ in the promoter of *ICS1* to positively modulate SAR-related SA production (Zhang et al., 2010a). Gene expression profiling revealed that 5’GAAATT3’ motifs were significantly over-represented in the promoters of SARD1 and CBP60g putative target genes (Truman and Glazebrook, 2012). SARD1 and CBP60g are functionally partially redundant (Zhang et al., 2010b; Wang et al., 2011). The upstream factor(s) that regulates the expression of *SARD1* and *CBP60g* have yet to be identified and the regulatory mechanism of SA biosynthesis during leaf senescence remains unknown.

Leaf senescence is a genetically programmed cell suicide process that is accompanied by mobilization of nutrients released during cell attrition to active growing regions, seeds or trunks (Gan and Amasino, 1997; Guo et al., 2021). The regulation of senescence is rather complex, and it involves activation of thousands of senescence-associated genes (*SAG*s) and/or inactivation of many senescence-down-regulated genes (Guo et al., 2004; Guo and Gan, 2012). TFs have been shown to have critical roles in regulating *SAG* expression and leaf senescence. For example, *AtNAP*, a NAC family TF gene, acts as a master regulator of leaf senescence because *atnap* null mutants display a 10-day delay in leaf senescence whereas its inducible expression in young leaves readily causes precocious senescence (Guo and Gan, 2006). The role of NAP orthologues in leaf senescence has been demonstrated in wheat (Uauy et al., 2006), maize (Zhang et al., 2012b), rice (Liang et al., 2014), cotton (Fan et al., 2015), peach (Li et al., 2016), and cabbage (Li et al., 2021). *NAP* also has a major role in senescence of rose petals (Zou et al., 2021) and Arabidopsis fruits (Kou et al., 2012). The direct target genes of *AtNAP* are of significant interest for understanding the molecular circuitry of leaf senescence regulation. It is known that AtNAP TF directly binds to the promoter of *SAG113* to activate the expression of a senescence-specific and Golgi-localized protein phosphatase 2C gene to promote senescence (Zhang and Gan, 2012; Zhang et al., 2012a). The TF also physically binds to the promoter of cytokinin oxidase genes in Arabidopsis, rice (Hu et al., 2021) and rose (Zou et al., 2021) to degrade the senescence-retardant cytokinins, which facilitates the senescence process.

Here we report that a senescence up-regulated gene named *SAG202* (At1G73805) is a direct target gene of *AtNAP*; sequence analysis reveals that *SAG202* is identical to *SARD1*. AtNAP physically binds to the promoter region of *SAG202*, but does not bind to *CBP60g*, and *SAG202* binds to the promoter region of *ICS1* (but not *ICS2*), as revealed by yeast one-hybrid experiments. Knockouts of *SAG202* and *ICS1* have lower levels of SA and display a significant delay in leaf senescence whereas inducible overexpression of *SAG202* leads to high levels of SA and premature leaf senescence. Quantitative PCR analyses further reveal that elevated SA levels can feedback up-regulate *AtNAP* and *SAG202*. These findings suggest that there is a unique feedback regulatory loop consisting of SA-*AtNAP*-*SAG202*-*ICS1*-SA that modulates the SA biosynthesis to control leaf senescence in Arabidopsis.

## Results

### *SAG202* is up-regulated during leaf senescence

**Fig. 1:**
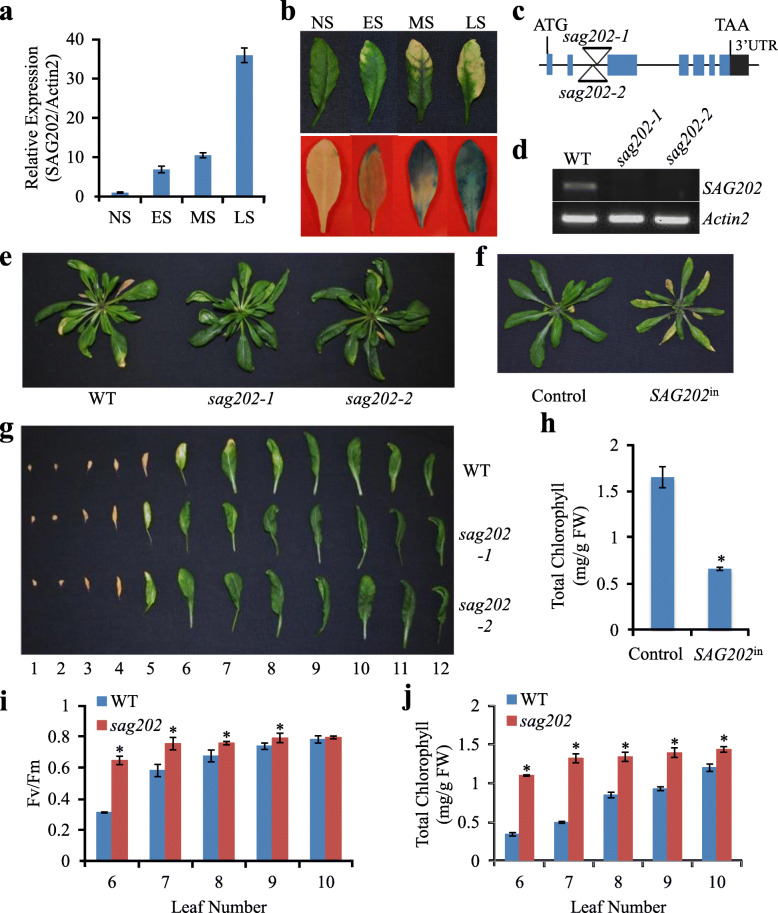
Phenotypic and molecular analyses of *SAG202* in Arabidopsis. (**a**) qPCR analysis of the transcript levels of *SAG202*/*SARD1* in WT leaves at different developmental stages. NS, fully expanded non-senescing stage; ES, early senescence stage (< 25% yellowing); MS, mid-senescence stage (~ 50% yellowing); LS, late senescence stage (> 75% yellowing). Relative expression levels were calculated and normalized with respect to *Actin 2* (*ACT2*) transcripts. Error bars indicate SD of three biological repeats. (**b**) GUS staining of the fifth leaves from *P*_*SAG202*_-*GUS* transgenic plants at different senescing stages. (**c**) Diagram of the T-DNA insertion locations of two *sag202* mutants. (**d**) The expression of *SAG202* is knocked out in the mutants shown in C as revealed by RT-PCR. (**e**) Age-matched 35 DAG WT and *sag202* null mutants. DAG, days after germination. (**f**) Precocious leaf senescence in *SAG202*-inducible expression line (*SAG202*^in^ or 8004/7001) (photo was taken 4 days after DEX induction). (**g**) Leaves detached from the age-matched 35 DAG plants in e (counted from bottom with the oldest leaf as 1 and the youngest leaf as 12). (**h**) The chlorophyll contents of the fifth and sixth leaves from control (containing pTA7001 only) and *SAG202*^in^ lines. (**i**, **j**) Chlorophyll contents and *F*_v_/*F*_m_ of the sixth to tenth rosette leaves of the age-matched 35 DAG plants from WT and the *sag202* mutants. Error bars indicate SD of three biological repeats. *P < 0.05 using Student’s t-test

*SAG202* (At1G73805) was initially identified during our analysis of the Arabidopsis leaf senescence transcriptome (Guo et al., 2004) and was later reported as *SARD1* (Zhang et al., 2010b; Wang et al., 2011) (hereafter *SAG202* is used). The transcript levels of *SAG202* were examined in leaves at different senescence stages using qPCR (Fig. 1a). To further investigate the expression pattern of *SAG202*, the *GUS* reporter gene was fused to the 3’ end of a 2.2-kb region of the *SAG202* promoter (P_*SAG202*_). The GUS staining patterns of the rosette leaves from P_*SAG202*_*-GUS* transgenic Arabidopsis showed that *SAG202* was expressed quite specifically in senescing leaves (Fig. 1b).

### Leaf senescence is significantly delayed in *sag202* knockout mutants and precociously accelerated in *SAG202* inducible overexpression lines

Two T-DNA lines, namely *sag202–1* (SALK_052422) and *sag202–2* (SALK_138476C) (Fig. 1c) in which *SAG202* was knocked out (Fig. 1d), were used to investigate the role of *SAG202* in leaf senescence. Compared with the wild type (WT), both knockout lines displayed a significant delay in leaf senescence phenotypically (Fig. 1e, g, and Supplementary Fig. S1) and physiologically (Fig. 1i, j). Because both knockout lines had the same phenotype, only *sag202–1* was used in the following experiments and referred to as *sag202* for simplicity.

**Fig. 2:**
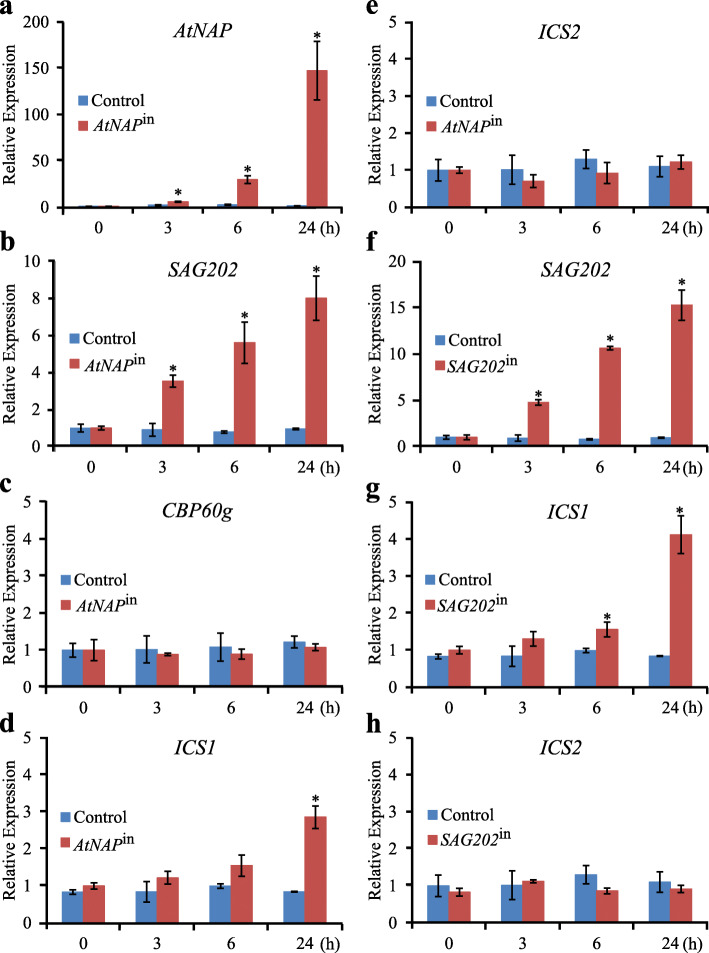
qPCR analyses of gene expression upon chemical induction of *AtNAP* or *SAG202*. (**a**-**e**) The transcript levels of *AtNAP*, *SAG202*/*SARD1*, *CBP60g*, *ICS1* and *ICS2* in the AtNAP inducible (*AtNAP*^in^) and control lines at 0 h, 3 h, 6 h and 24 h after DEX treatment. (**f**-**h**) The transcript levels of *SAG202*, *ICS1* and *ICS2* in the *SAG202*^in^ and control lines at 0 h, 3 h, 6 h and 24 h after DEX treatment. Relative expression levels were calculated and normalized with respect to *Actin 2* (*ACT2*) transcripts. Error bars indicate SD of three biological repeats. *P < 0.05 using Student’s t-test

The role of *SAG202* in leaf senescence was also investigated in dexamethasone (DEX) inducible gain-of-function lines harboring both pTA7001 and pGL8004 constructs. pTA7001 (control) provides constitutive expression of a recombinant transcription factor (TF), GAL4^BD^-VP16^AD^-GR, in transgenic plants (Guo and Gan, 2006). In pGL8004 construct, *SAG202* is driven by a promoter containing six tandem copies of the GAL4 upstream activation sequence. When DEX (a synthetic glucocorticoid) binds to GR and causes conformational changes, VP16 is able to activate transcription of *SAG202* in plants harboring both pTA7001 and pGL8004 (*SAG202*^in^). As shown in Fig. 1f and h, treatment of 20-day-old non-senescing plants with 30 μM DEX caused precocious leaf senescence in *SAG202*^in^ lines but not in the control lines. qPCR analyses showed that *SAG202* was strongly induced in *SAG202*^in^ lines but not in the control lines (Fig. 2f).

### *SAG202* (but not *CBP60g*) and *ICS1* (but not *ICS2*) are co-induced with *AtNAP*

*AtNAP* is a NAC family TF that is up-regulated during senescence, and its DEX-inducible expression lines (*AtNAP*^in^) are readily available (Guo and Gan, 2006). Upon DEX treatment, the expression of *AtNAP* was significantly induced in *AtNAP*^in^ lines but not in control plants (Fig. 2a). qPCR analyses revealed that *SAG202* and *ICS1* were also induced (Fig. 2b, d), but *CBP60g*, a gene closely related to *SAG202*, and *ICS2* were not induced (Fig. 2c, e). *ICS1* expression was co-induced with the induction of *SAG202* in *SAG202*^in^ lines while the expression of ICS2 was not induced (Fig. 2g, h).

### AtNAP TF physically binds to the promoter region of *SAG202* (but not *CBP60g*) in yeast cells and *in planta*

**Fig. 3:**
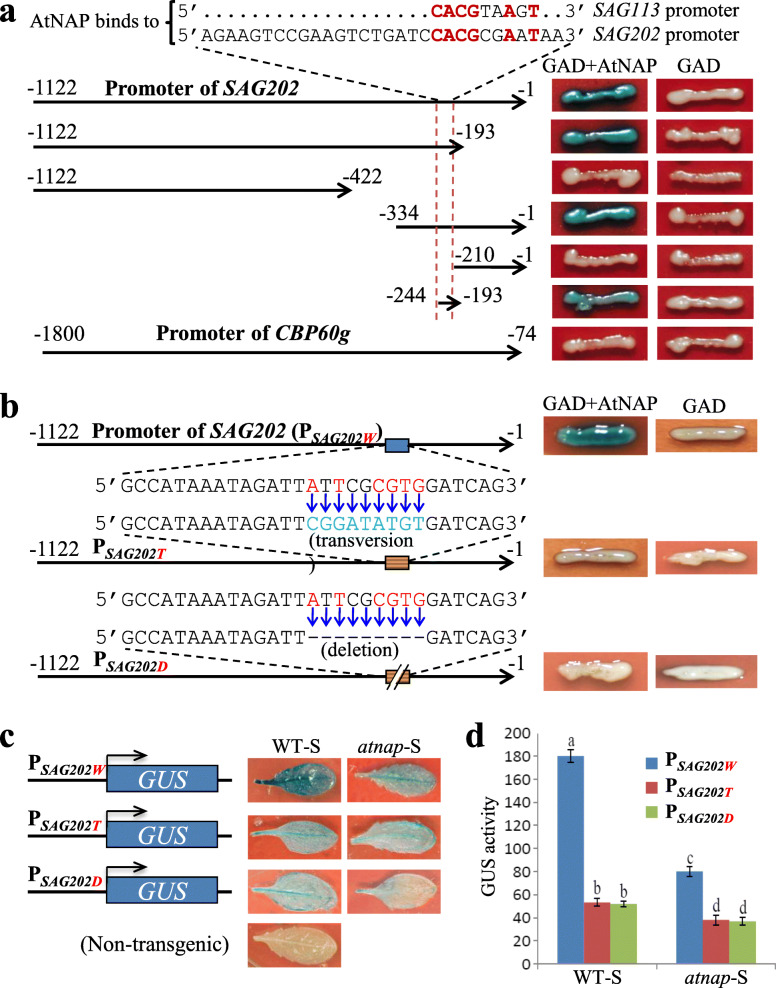
Binding of *AtNAP* to the *SAG202* promoter truncations and mutants in yeast and *in planta*. (**a**) Binding of AtNAP to the *SAG202* promoter truncations revealed by yeast one-hybrid assay. The *LacZ* reporter gene driven by various *SAG202* promoter truncations was used to test the binding ability of the GAD-AtNAP fusion protein. Red dash lines indicate promoter sequence that is highly conserved to the 9-bp (red letters) AtNAP binding site of the *SAG113* promoter (Zhang and Gan, 2012). The immediate upstream bp of the translation start site was numbered as − 1. The *CBP60g* promoter (1727 bp in length) was also tested. (**b**) Failure of AtNAP binding to the *SAG202* promoter that contains either transversion mutation or deletion of the motif sequence in yeast cells. (**c**) GUS staining of senescent leaves in transgenic Arabidopsis plants (WT or *annap* mutant background) harboring P_*SAG202W*_-*GUS* (_*W*_ for WT), P_*SAG202T*_-*GUS* (_*T*_ for transversion; the promoter contains the transversion mutation shown in (**b**)) or P_*SAG202D*_-*GUS* (_*D*_ for deletion; the promoter contains the deletion mutation shown in (**b**)). (**d**) GUS enzymatic activities (expressed as nmol methylumbelliferone produced min ^− 1^ mg ^− 1^ protein) of senescent leaves of transgenic plants shown in (**c**). Data are mean values ± SD of five samples. Significant (*P* > 0.05) differences between means are indicated by different letters. ANOVA analysis with LSD test was used

The above co-induction of *SAG202* with *AtNAP* raised the possibility of *SAG202* being a direct target gene of *AtNAP*. To test this, we performed yeast one-hybrid experiments in which a series of truncated promoter fragments of *SAG202* (Fig. 3a) were cloned in front of a *LacZ* reporter gene as promoter baits to form various reporter constructs; the *AtNAP* coding sequence was fused with the yeast GAL4 activation domain (GAD) to form the effector GAD-AtNAP construct (Zhang and Gan, 2012). The AtNAP TF was able to physically bind to a specific region of *SAG202* promoter containing 5’CACGcgAaT3’ that is very similar to the 9-bp *AtNAP* core binding sequence, 5’CACGtaAgT3’ (nucleotides in lower case are variable), in the promoter of *SAG113* (Zhang and Gan, 2012) (Fig. 3a). In contrast, AtNAP TF did not bind to the promoter of *CBP60g* (Fig. 3a). The binding of AtNAP TF to the motif 5’CACGcgAaT3’ on the *SAG202* promoter (P_*SAG202*_) in yeast cells was confirmed by transversion and deletion mutants of the sequence: when mutated, AtNAP TF could no longer bind to the promoter (Fig. 3b).

The physical interaction of AtNAP TF to P_*SAG202*_ was further examined *in planta*. The P_*SAG202W*_ (wild-type promoter) and its variants with either transversion (P_*SAG202T*_) or deletion mutation (P_*SAG202D*_) shown in Fig. 3b were fused with the *GUS* reporter gene and transformed into WT or *atnap* null background. The GUS activities in senescent leaves (WT-S or *atnap*-S) were analyzed by histochemical staining and enzymatic assay, which revealed that the GUS activities with either of the mutated promoter motif in senescent leaves of WT were reduced to less than 30% of the P_*SAGW*_-*GUS* plants and that the GUS activities in the *atnap* null background were less than 45% of the activities in WT background (Fig. 3c,d). The data further supported the AtNAP TF physically bound to the motif 5’CACGcgAaT3’ of the *SAG202* promoter to direct the gene expression *in planta*.

### SAG202 TF physically binds to the promoter of *ICS1* (but not *ICS2*) in yeast cells and *in planta*

**Fig. 4:**
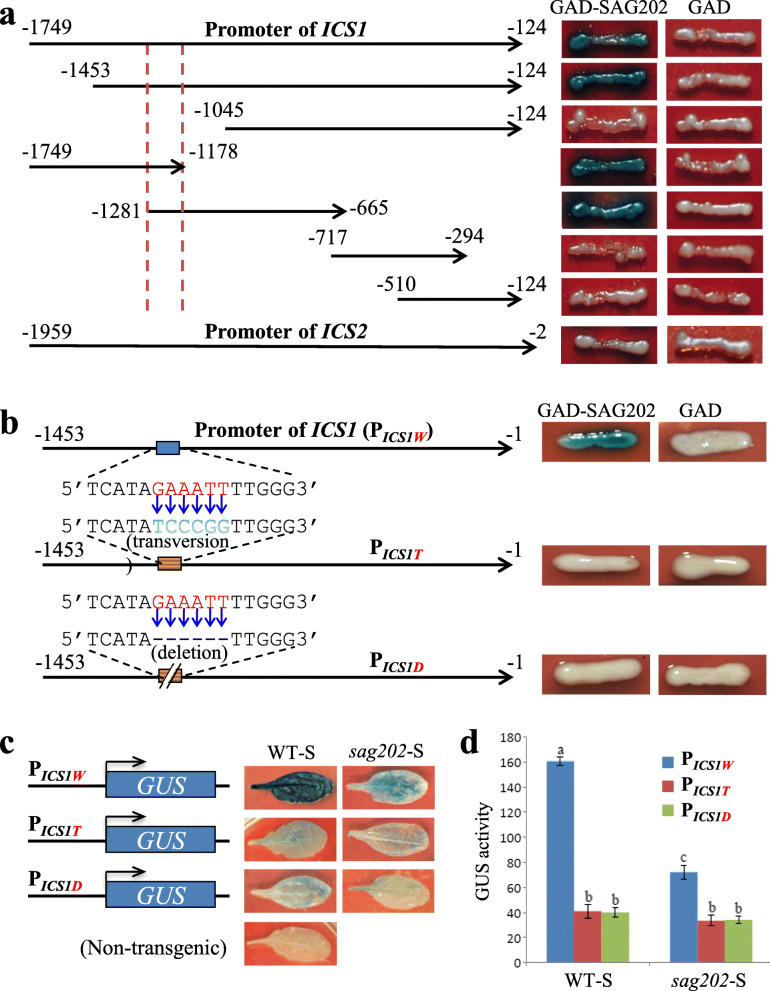
Binding of *SAG202* to the *ICS1* promoter truncations and mutants in yeast and *in planta*. (**a**) Binding of *SAG202* to the *ICS1* promoter truncations. The *LacZ* reporter gene driven by various *ICS1* promoter truncations was used to test binding ability of the GAD-SAG202 fusion protein. Red dash lines indicate the 104 bp promoter region containing the *SAG202* binding sequence. The *ICS2* promoter (1626 bp in length) was also tested. (**b**) Failure of SAG202 binding to the *ICS1* promoter that contains either transversion mutation or deletion of the motif sequence GAAATT (red letters) within the 104 bp region in yeast cells. (**c**) GUS staining of senescent leaves in transgenic Arabidopsis plants (WT or *sag202* mutant background) harboring P_*ICS1W*_-*GUS* (_*W*_ for WT), P_*ICS1T*_-*GUS* (_*T*_ for transversion; the promoter contains the transversion mutation shown in **b**) or P_*ICS1D*_-*GUS* (_*D*_ for deletion; the promoter contains the deletion mutation shown in **b**). (**d**) GUS enzymatic activities (expressed as nmol methylumbelliferone produced min ^− 1^ mg ^− 1^ protein) of senescent leaves of transgenic plants shown in **c**. Data are mean values ± SD of five samples. Significant (*P* > 0.05) differences between means are indicated by different letters. ANOVA analysis with LSD test was used

To investigate if the SAG202 TF physically interacts with the *ICS1* promoter, various *ICS1* promoter fragments with truncations were generated and yeast one-hybrid system was utilized to show that the SAG202 TF was indeed able to bind to a 104 bp region (− 1178 ~ − 1281) of the promoter of *ICS1*. The 1958-bp promoter of *ICS2* (− 2 ~ − 1959) was also used in the yeast one-hybrid experiment, which revealed there was no physical interaction between the SAG202 TF and the *ICS2* promoter (Fig. 4a). The 104 bp region contained a 6 bp motif 5’GAAATT3’ that was believed to be the binding site of *SAG202*. To test this, *ICS1* promoters with either transversion or deletion mutation were used in the yeast one-hybrid experiments, and the mutations abolished the binding of SAG202 to the *ICS1* promoter variants in the yeast cells (Fig. 4b). Further analyses involving the use of the *ICS1* promoters with or without the transversion or deletion mutation to direct the *GUS* expression in WT and *sag202* null background revealed that SAG202 also physically interacted with the 5’GAAATT3’ *cis* element of the *ICS1* promoter *in planta* (Fig. 4c,d).

### Both *AtNAP* and *SAG202* are positively regulated by SA

**Fig. 5:**
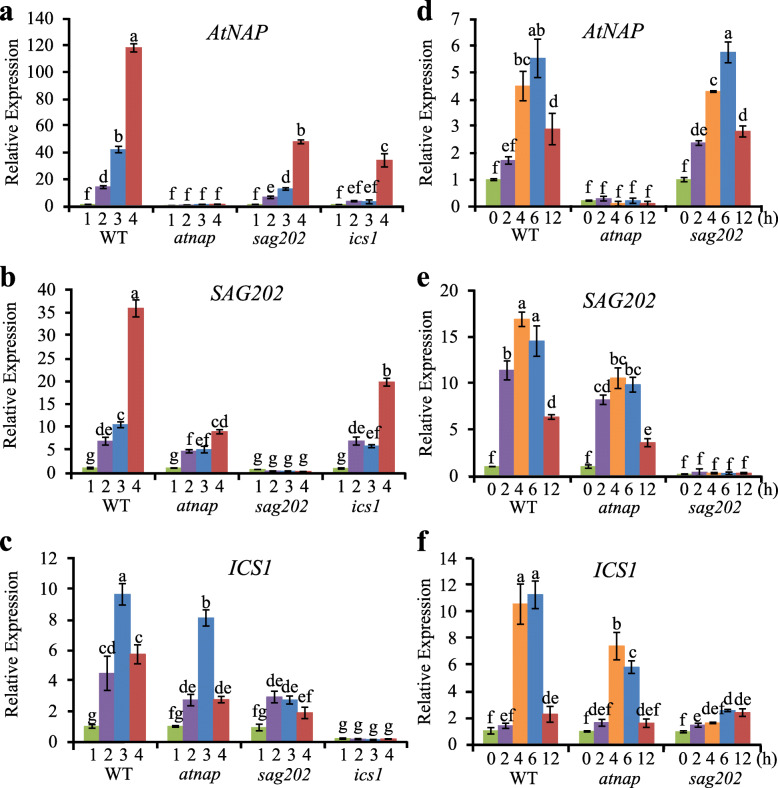
qPCR analyses of transcript levels of *AtNAP*, *SAG202*, and *ICS1* in different mutants during senescence or after SA treatment. (**a**-**c**) Transcript levels of *AtNAP*, *SAG202* and *ICS1* in leaves of WT, *atnap*, *sag202* and *ics1* null mutants at different senescence stages (1, NS; 2, ES; 3, MS; 4, LS as described in legend to Fig. 1a). (**d**-**f**) Transcript levels of *AtNAP*, *SAG202* and *ICS1* in NS leaves of WT, *atnap* and *sag202* null mutants at time points after treatment with 5 mM SA. Relative expression levels were calculated and normalized with respect to *Actin 2* (*ACT2*) transcripts. Error bars indicate SD of three biological repeats. Significant (*P* < 0.05) differences between means are indicated by different letters using Tukey’s HSD test

The above data revealed a regulatory chain consisting of *AtNAP*-*SAG202*-*ICS1* operating to produce SA during leaf senescence. If so, knocking out of an upstream gene should effect the expression of its downstream gene(s). We thus performed qPCR to analyze the expression levels of these genes in WT, *atnap*, *sag202* and *ics1* null mutants at different senescence stages (Fig. 5a-c). As expected, the transcript levels of both *SAG202* and *ICS1* were significantly reduced in the absence of *AtNAP* (Fig. 5b, c); similarly, the *ICS1* expression levels were remarkably lowered in the *sag202* mutants (Fig. 5c). The expression levels of *CBP60g* and *ICS2*, two genes outside of the regulatory chain, were not altered in any of the mutant backgrounds (Supplementary Fig. S2).

Interestingly, the expression levels of *AtNAP* in either *sag202* or *ics1* null mutants were reduced (Fig. 5a), and the transcript levels of *SAG202* in leaves at the mid-senescence stage (MS) in the *ics1* background were also decreased (Fig. 5b). These data suggested the possibility that the end product SA of the regulatory chain might feedback regulate those genes. To test this hypothesis, we analyzed the expression levels of *AtNAP*, *SAG202*, *ICS1* in WT, *atnap* and *sag202* mutants upon SA treatments. *AtNAP* (Fig. 5d) and *SAG202* (Fig. 5e) were significantly induced by SA whereas the induction of *ICS1* in the *sag202* null mutants was not as significant (Fig. 5f), suggesting that *AtNAP* and *SAG202* were positively feedback regulated by SA.

### Free SA levels were reduced in *atnap* and *sag202* mutants and elevated in *AtNAP*^in^ and *SAG202*^in^ lines

**Fig. 6:**
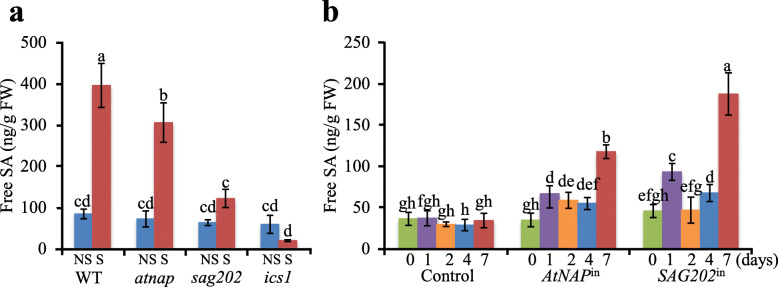
LC-MS/MS analyses of free SA levels in WT, *atnap*, *sag202*, *ics1* null mutants, *AtNAP*^in^ and *SAG202*^in^ lines. (**a**) Free SA levels in NS and S (~ 50% yellowing) leaves of WT, *atnap*, *sag202* and *ics1* mutants, respectively. (**b**) Free SA levels in young leaves of *AtNAP* inducible lines (*AtNAP*^in^) and *SAG202* inducible lines (*SAG202*^in^) at 0, 1, 2, 4, and 7 days after DEX induction. Error bars indicate SD of three biological repeats. Significant (*P* < 0.05) differences between means are indicated by different letters using Tukey’s HSD test

The free SA levels in fully expanded non-senescing leaves (NS) and senescing leaves (S) of WT, *atnap*, *sag202* and *ics1* mutants were quantitatively analyzed using LC-MS/MS. The SA levels in the senescing leaves were significantly reduced in these null mutants but remained unchanged in the non-senescing leaves of any of the plants (Fig. 6a).

The free SA levels in leaves with induced expression of *AtNAP* (*AtNAP*^in^) or *SAG202* (*SAG202*^in^) were also quantitated. As shown in Fig. 6b, the SA levels were significantly increased readily one day after the DEX induction.

### The *SAG202-ICS1*-SA regulatory chain is shared between leaf senescence and defense response

**Fig. 7:**
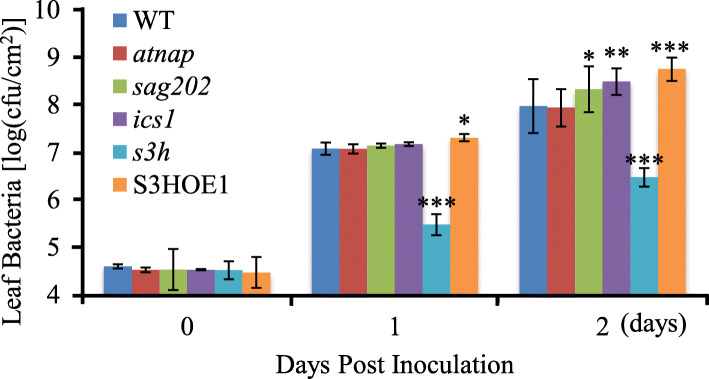
Growth of *P. syringae pv.* Tomato DC3000 in leaves of WT, *atnap*, *sag202*, *ics1*, *s3h* null mutants and S3HOE1 transgenic plants. The number of colony-forming units (cfu) per square centimeter of leaf area was determined 0, 1, and 2 days after inoculation. Error bars indicate SD of three biological repeats. *, ** and *** indicate *P* < 0.05, *P* < 0.01 and *P* < 0.001, respectively. Student’s t-test was used. The *s3h* null mutant (resistant to the infection) and S3HOE1 transgenic plants (susceptible to the infection) (Zhang et al., 2013) were included for comparison purpose

**Fig. 8:**
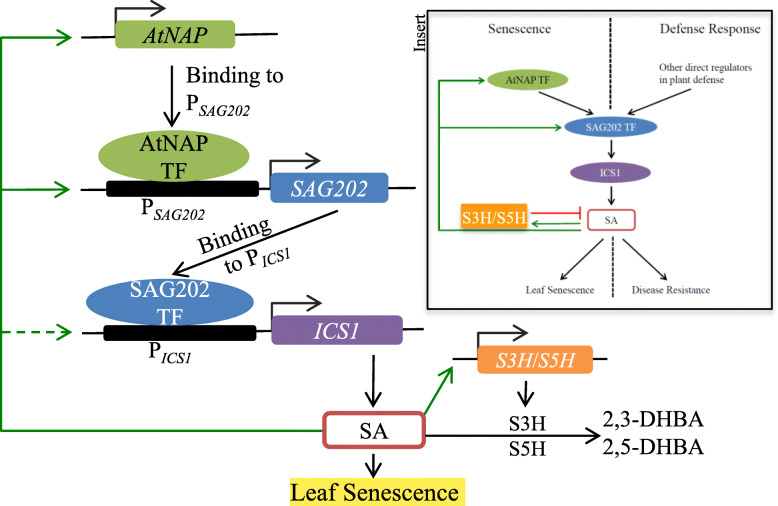
A working model of SA-*AtNAP*-*SAG202*-*ICS1*-SA positive feedback loop in leaf senescence and its convergence/divergence in defense response in Arabidopsis. At the onset of and during leaf senescence, AtNAP TF physically binds to the promoter of *SAG202* to direct the target gene expression. Subsequently the SAG202 TF activates its direct target gene *ICS1* that is involved in the SA biosynthesis. The produced SA in turn feedback upregulates both *AtNAP* and *SAG202*. When the SA levels increase to a threshold, *S3H* (encoding an SA 3-hydroxylase) and *S5H* is induced to prevent overaccumulation of SA (Zhang et al., 2013; Zhang et al., 2017b). Too high levels of SA will cause hypersensitive response (HR)-like fast cell death. Leaf senescence is a slow programmed cell death process to allow nutrients released from degradation of proteins and other macromolecules to be recycled to active growing region or storage organs. **Insert**: A diagram showing convergence and divergence between leaf senescence and defense response with regard to the newly uncovered SA-*AtNAP*-*SAG202*-*ICS1*-SA regulatory loop. SA-*AtNAP*-*SAG202*-*ICS1*-SA feedback loop is activated to modulate endogenous SA levels in leaf senescence but not in defense responses. In defense response part of the loop, *SAG202*-*ICS1*-SA is activated

Our studies showed that the positive feedback regulatory loop consisting of SA-*AtNAP*-*SAG202*-*ICS1*-SA operates during leaf senescence, and the *SAG202*-*ICS1* node has been clearly shown to function in plant defense response (Wildermuth et al., 2001; Zhang et al., 2010b; Wang et al., 2011). To investigate whether *AtNAP* also has any roles in disease resistance, we inoculated mature non-senescing leaves of *atnap*, *sag202*, *ics1* mutants and WT with *Pseudomonas syringae* pv. Tomato DC3000 and found that the pathogen resistance in the *atnap* mutant was not changed compared with that in WT while the *sag202* and *ics1* mutants became more susceptible to the pathogen infection (Fig. 7). These data strongly suggest that the *SAG202*-*ICS1*-SA regulatory chain is shared by leaf senescence and defense response and that the up-stream component *AtNAP* appears to be leaf senescence specific (Fig. 8).

## Discussion

Because of the significant role of SA in plant defense, much research has been performed to decipher its biosynthesis and signaling in plant (Shirasu et al., 1997; Dangl, 1998; Fu et al., 2012). There are two pathways leading to the production of SA in plants: one from phenylalanine and the other from chorismate via isochorismate (IC) (Dempsey et al., 2011). In Arabidopsis, the IC pathway contributes predominantly to SA accumulation during defense responses and *isochorismate synthase 1* (*ICS1*) has the major role in this accumulation (Wildermuth et al., 2001). The IC pathway appeared to be predominant during leaf senescence in Arabidopsis because the SA levels in senescent leaves of *ics1* were less than 5% of WT (Fig. 6a). Further studies showed that SARD1 and CBP60g bind to the promoter of *ICS1* to regulate this gene’s expression (Zhang et al., 2010b; Wang et al., 2011). Which TFs regulate *SARD1* (and CBP60g) is unknown. Our research addressed this question by identifying AtNAP (a NAC family TF) as a direct upstream regulator of *SARD1*(Fig. 8); this was supported by at least three lines of evidence: (i) the yeast one-hybrid experiments showed that AtNAP could physically bind to a promoter region of *SAG202* (identical with *SARD1*) that contains a highly conserved sequence to which AtNAP binds (Fig. 3a, b), (ii) AtNAP binds to the 9 bp motif of the SAG202 promoter in planta (Fig. 3c, d), and (iii) *SAG202* was co-induced when *AtNAP* was chemically induced (Fig. 2b). Interestingly, *CBP60g*, the close homolog of *SAG202,* is unlikely to be directly regulated by *AtNAP* because AtNAP could not bind to the promoter region of *CBP60g* (Fig. 3a) and that *CBP60g* was not co-induced with *AtNAP* (Supplementary Fig. S2). In addition to uncovering the *AtNAP*-*SAG202* chain, we also provided new lines of evidence that *SAG202* physically binds to the promoter of *ICS1* (but not *ICS2*) as shown by our yeast one-hybrid experiment results (Fig. 4a, b), by *in planta* analyses (Fig. 4c, d), and by induction of the expression of *ICS1* (Fig. 2d, g) but not *ICS2* (Fig. 2e, h) through chemical activation of *AtNAP* or *SAG202*. SAG202 TF/SARD1 has been previously shown to directly regulate *ICS1* using chromatin immunoprecipitation (ChIP), electrophoretic mobility shift assay (EMSA) and promoter sequence analysis (Zhang et al., 2010b; Truman and Glazebrook, 2012). These data reveal a unique regulatory chain consisting of *AtNAP*-*SAG202*-*ICS1* (Fig. 8), which significantly advanced our understanding of molecular regulatory mechanism of the SA biosynthesis.

It is known that the SA levels are higher in senescing leaves than in non-senescing leaves and SA has an important role in controlling leaf senescence in Arabidopsis (Morris et al., 2000; Zhang et al., 2013; Zhang et al., 2017b; Yu et al., 2021). *SARD1*-*ICS1* was shown to contribute to the SA production during defense responses (Wildermuth et al., 2001; Zhang et al., 2010b; Wang et al., 2011), but whether it, together with its upstream component *AtNAP*, also operates and functions during leaf senescence was not known. This research provided several lines of evidence that the regulatory chain operates in leaf senescence. The first line of evidence comes from the qPCR analysis of transcript levels of individual genes in the chain. As shown in Fig. 2a-d, the expression levels of *AtNAP*, *SAG202* and *ICS1* were all up-regulated upon chemical induction of *AtNAP*. The second line of evidence is from the quantification of SA levels in senescing leaves of respective null mutants. When individual genes of the regulatory chain were knocked out, the SA levels in senescing leaves were all significantly reduced (Fig. 6a). It should be noted that there is still ~ 30% SA in the senescent leaves of *sag202* and ~ 75% in *atnap* (Fig. 6a), which could be due to *ICS2* that is upregulated during senescence (Fig. S2) and/or *ICS1* that is activated by such retrograde signaling protein WHIRLY1 (Lin et al., 2020) and senescence-associated TFs as WRKY75, WRKY51, WRKY28, WRKY55 and WRKY46 that have been shown to directly bind to the promoter of *ICS1* (Guo et al., 2017; Zhang et al., 2017a; Tian et al., 2020; Wang et al., 2020).

In the absence of either *SAG202* or *ICS1*, the transcript levels of *AtNAP* were significantly reduced in senescing leaves, in particular late-senescence (LS) leaves (Fig. 5a). Similarly, the expression levels of *SAG202* in the *ics1* null background were also decreased (Fig. 5b). These data suggested that SA, the end product of the regulatory chain, might positively feedback regulate *AtNAP* and *SAG202* as shown in Fig. 8. This feedback regulation is supported by the fact that exogenous SA markedly elevated the *AtNAP* transcript levels in the *sag202* mutants (and WT) (Fig. 5d). In the absence of *AtNAP*, external SA was able to highly induce the *SAG202* expression (Fig. 5e), suggesting that SA may have a positive feedback regulation on *SAG202* beyond *AtNAP*. In contrast, in the absence of *SAG202*, the *ICS1* expression levels were not significantly altered by the external SA (Fig. 5f), indicating that *ICS1* is unlikely to be positively feedback regulated by SA.

Previous studies suggested an important role of SA in leaf senescence (Morris et al., 2000; Gan, 2010; Zhang et al., 2013; Zhang et al., 2017b). Examples include the observations that there are higher levels of SA in senescing leaves compared with those in non-senescing leaves, leaf senescence is delayed in NahG or S3HOE plants in which a SA-degrading enzyme of bacterial or Arabidopsis origin is overexpressed, and the leaf senescence is accelerated in the *s3h* null plants in which SA are over-accumulated (Zhang et al., 2013). In this research, we found that when any of the genes in the regulatory loop are knocked out, the endogenous SA levels are significantly reduced (Fig. 5a) and the leaf longevity is substantially extended (Fig. 1a-b, Supplementary Fig. S1). Conversely, when *AtNAP* and *SAG202* were individually chemically induced, the endogenous SA levels were enhanced (Fig. 6b) and the plants displayed precocious leaf senescence (Fig. 1f, h). These data reinforce SA’s role in promoting leaf senescence.

The shared regulatory chain of *SAG202*/*SARD1*-*ICS1*-SA is initiated by different cues and regulated to different extent of SA accumulation (insert in Fig. 8). The upstream regulator of *SAG202* during disease resistance is not known yet, while in leaf senescence, *SAG202* is regulated by AtNAP TF. *CBP60g*, the closely related family member to *SAG202*, was not directly regulated by AtNAP TF; however, *CBP60g* might also have a role in leaf senescence because its expression profile showed a senescence-associated elevation (Supplementary Fig. S2). In defense response, both *CBP60g* and *SAG202* are involved in the induction of SA; after pathogen infection, SA level was elevated to a very high level in local leaves and leaded to a suicide cell death (Raskin, 1992; Zhang et al., 2010b). However, in age-dependent leaf senescence, SA level in senescing leaves was up-regulated to about 4 times higher than that in non-senescing leaves (Morris et al., 2000; Zhang et al., 2013; Zhang et al., 2017b) and resulted in a gentle and slow programmed cell death (PCD) necessary for remobilization of nutrients released during senescence to active growing tissues or storage organs such as seeds and trunk (Gan and Amasino, 1997).

Another difference is the significance of *ICS1* in SA production between disease resistance and leaf senescence. SA level was almost undetectable in senescing leaves of *ics1* mutants, which was even lower than its level in non-senescing leaves (Fig. 6a). This can be interpreted that *ICS1* contributes to almost all SA production in senescing Arabidopsis leaves. In plant defense responses, however, there were still SA production in *ics1* mutants, suggesting that there are other genes such as *ICS2* (Garcion et al., 2008) or other SA biosynthesis pathways such as PAL pathway (Dempsey et al., 2011) functions in the SA biosynthesis. In addition to the regulation of SA anabolism, the level of SA is regulated by SA catabolism during leaf senescence. Studies showed that SA 3-hydroxylase (S3H) and SA 5-hydroxylase (S5H) are induced by SA and converts SA to its inactive forms 2,3-DHBA and 2,5-DHBA, respectively, which constitutes the negative feedback regulation of SA in leaf senescence to prevents SA over accumulation (Zhang et al., 2013; Zhang et al., 2017b).

## Materials and methods

### Plant materials and growth conditions

*Arabidopsis thaliana* ecotype Columbia was used in this study. The *atnap* knockout mutants, the *AtNAP*-inducible expression lines (Guo and Gan, 2006), and two T-DNA insertion lines (SALK_052422 and SALK_128476C, from ABRC) were all in the Columbia background. Per http://signal.salk.edu/tdnaprimers.2.html, a PCR-based method was used to identify homozygous T-DNA insertion mutants. The T-DNA left border primer G2325 (LBb1.3) and the gene-specific primers, G3832 and G3833 for *sag202–1* (SALK_052422) and G3809 and G3810 for *sag202–2* (SALK_128476C), were used. Plants homozygous for the T-DNA insertion were used in this study. All primers used in this research are listed in Supplemental Table S1.

Seed sterilization and growth were as previously described (Guo and Gan, 2006). The mutants, transgenic plants, and WT were grown side by side.

### Plasmid construction

For the P_*SAG202*_*-GUS* construct (pGL8002), a 2201-bp promoter fragment of *SAG202* (At1G73805) was amplified from Arabidopsis genomic DNA by PCR with primers G3830 and G3831, cloned into pGEM-T easy vector (Promega, Madison, USA), sequenced, digested with *Pst I* and *Nco I* and inserted into pBI211 to form pGL8002.

To generate DEX-inducible *SAG202* overexpression construct (pGL8004), the 1357-bp full length CDS of *SAG202* was amplified from Arabidopsis cDNA by PCR with primers G3828 and G3829, ligated to pGEM-T easy vector, sequenced, digested with *Hind III* (Klenow fill-in) and *Pst I*, and cloned into the inducible binary vector pGL1152 (Guo and Gan, 2006) that was digested with *Spe I* (Klenow fill-in) and *Pst I* to form pGL8004.

Yeast one-hybrid assay-related constructs: pGL3175 (for producing GAD-AtNAP fusion protein in yeast) was constructed as described previously (Zhang and Gan, 2012). To construct pGL8040 (for producing GAD-SAG202 fusion protein in yeast), the *SAG202* coding sequence was amplified from Arabidopsis cDNA by PCR with primers G4020 and G3992, ligated to pGEM-T easy vector, sequenced, digested with *HindIII* and *XhoI*, and cloned into the pJG4–5 (Lin et al., 2007) to form pGL8040. To construct P_*SAG202*_*-LacZ*, *P*_*ICS1*_*-LacZ* reporter genes, the 1122-bp *SAG202* promoter (P_*SAG202*_) region and the 1625-bp *ICS1* promoter region (P_*ICS1*_) were amplified from the Arabidopsis genomic DNA. The pairs of primers used were G3967 and G3918 for P_*SAG202*_, and G3993 and G3994 for *P*_*ICS1*_. The amplified fragment was ligated to the pGEM-T easy vector, sequenced, then released from the plasmid with *EcoR I-Sal I* and *EcoR I-Xho I*, respectively, and cloned into pLacZi-2 μ (Lin et al., 2007) that was digested with *EcoRI-XhoI* to form pGL8017 and pGL8036, respectively. Other *LacZ* reporter gene plasmids containing various truncated *SAG202*, *ICS1*, *CBP60g* and *ICS2* promoter regions were similarly constructed using the primers listed in Supplementary Table S1.

### Histochemical GUS staining, chlorophyll assay, and *Fv/Fm* assay

Histochemical GUS staining, chlorophyll assay, *F*_v_*/F*_m_ assay were performed as previously described (Zhang and Gan, 2012; Hou et al., 2013).

### qPCR analyses of transcripts

Total RNA extractions from Arabidopsis leaves and real-time PCR analyses were performed according to (Hou et al., 2013). cDNA was synthesized from 3 μg of total RNA (treated with RNase-free DNase; New England Biolabs, USA) at 42 °C with MV-Reverse Transcriptase (Promega, USA) (Hu et al., 2021). For qPCR, 1 μL of each diluted sample (40 folds) was used as a template in a 25-μL reaction. All qPCR reactions were performed on a Bio-Rad IQ-5 thermocycler with an annealing temperature around 55 °C. Cycle threshold values were determined by IQ-5 Bio-Rad software assuming 100% primer efficiency (Hu et al., 2021). Primers used for quantitative RT-PCR were listed in Supplementary Table S1. Three repetitions were performed for each combination of cDNA samples and primer pairs.

### Plant transformation

Various constructs in binary vectors were transferred into *Agrobacterium tumefaciens* strain ABI1 that were subsequently used to transform Col-0 via the floral-dip method (Clough and Bent, 1998). Approximately 30 antibiotics-resistant T1 transgenic lines for each transgene were selected; phenotypic analyses were performed in T2 or advanced generations. Homozygous plants were used in all experiments.

### Dexamthasone (DEX) treatments

The glucocorticoid treatments were performed as described by Guo and Gan (2006). 30 μm examethasone (DEX) was sprayed twice (once a day) to 2-week-old plants grown in pots. Photos were taken 2 days after the last spray.

### SA treatment and chemical induction of gene expression

WT Col-0 plants, *atnap* and *sag202* mutant plants (all 20 days old) were sprayed with 0.005% Silwet L-77 with or without 5 mM SA. Glucocorticoid treatments were performed as previously described (Guo and Gan, 2006). Twenty-day-old plants were sprayed with 30 μM dexamethasone (DEX, a synthetic glucocorticoid) containing 0.005% Silwet L-77. The 5th, 6th and 7th rosette leaves of each plant (counted from bottom) were collected for RNA extraction at different time points after the spray.

### Yeast one-hybrid assay

Yeast one-hybrid assays were performed as previously described (Zhang and Gan, 2012). pGL3175 (the *GAD-AtNAP* fusion gene) was co-transformed with different *LacZ* reporter constructs containing different lengths of the *SAG202* and *ICS1* promoter fragments into the yeast strain EGY48. Similarly, pGL8040 (the *GAD-SAG202* fusion gene) was co-transformed with different *LacZ* reporter constructs containing different lengths of the *ICS1* promoter fragments into the yeast stain EGY48. The transformants were grown on proper dropout plates containing 5-bromo-4-chloro-3-indolyl-β-D-galactopyranoside (X-Gal) for the blue color development.

### SA quantification

Non-senescing and mid-senescence leaves (0.1–0.3 g) of WT, *atnap*, *sag202*, *ics1*, and the leaves (also 0.1–0.3 g) of *AtNAP*^in^ lines, *SAG202*^in^ lines and control lines at different time points after chemical induction were collected and analyzed for free SA using an LC–MS/MS (Zhang et al., 2013).

### Bacterial growth assay

The bacterial strain *Pto* DC3000 suspension in sterile water (OD_600_ = 0.002) were infiltrated into 6th and 7th leaves of 4-week-old plants using a needleless syringe. For determination of bacterial growth in inoculated leaves, the leaf samples were collected shortly (0d), 1d or 2d after inoculation. Bacterial inoculum preparation, syringe injection and bacterial pathogen enumeration were performed according to previously described (Katagiri et al., 2002).

### Supplementary Information

**Additional file 1:** **Supplementary Figure S1.** Delayed leaf senescence phenotype in atnap, sag202/sard1 and ics1 null mutants; **Supplementary Figure S2.** qPCR analyses of transcript levels of CBP60g and ICS2 in leaves of WT, atnap, sag202 and ics1 null mutants at different senescence stages; **Supplementary Table S1.** Primers used in this research.

## Data Availability

The data and materials will be available upon reasonable request.

## Abbreviations

At: *Arabidopsis thaliana*
DEX: dexamethasone
EIL1: EIN3-like 1
EIN3: ethylene insensitive 3
GAD: GAL4 activation domain
IC: isochorismate
ICS1: isochorismate synthase 1
LAR: local acquired resistance
NAC: NAM (no apical meristem, Petunia), ATAF1–2 (*Arabidopsis thaliana* activating factor), and CUC2 (cup-shaped cotyledon, Arabidopsis)
LC-MS/MS: liquid chromatography with tandem mass spectrometry
NAP: NAC-LIKE, Activated BY AP3/P1
Pto: *Pseudomonas syringae* pv. *tomato*
S3H: SA 3-hydroxylase
S5H: SA 5-hydroxylase
SA: Salicylic Acid
SARD1: systemic acquired resistance deficient 1
SAGs: senescence-associated genes
SAR: systemic acquired resistance
TF: transcription factor
