# Supplementary material for: A positive feedback regulatory loop, SA-AtNAP-SAG202/SARD1-ICS1-SA, in SA biosynthesis involved in leaf senescence but not defense response
Source: Mol Hortic. 2022 Jun 17;2:15. doi: 10.1186/s43897-022-00036-x (PMC10515000; doi:10.1186/s43897-022-00036-x)
Supplement: Supplementary file 1 — Additional file 1: Supplementary Figure S1. Delayed leaf senescence phenotype in atnap, sag202/sard1 and ics1 null mutants; Supplementary Figure S2. qPCR analyses of transcript levels of CBP60g and ICS2 in leaves of WT, atnap, sag202 and ics1 null mutants at different senescence stages; Supplementary Table S1. Primers used in this research. [file 43897_2022_36_MOESM1_ESM.docx]

**Supplementary Information (SI)**

**Supplementary figures**

**Supplementary Figure S1 Delayed leaf senescence phenotype in *atnap*, *sag202*/*sard1* and *ics1* null mutants.** (A) Age-matched 40 DAG WT, *atnap*, *sag202* and *ics1* null mutants. (B) Phenotypes of leaves detached from the age-matched 40 DAG plants in A. DAG, days after germination.

**Supplementary Figure S2 qPCR analyses of transcript levels of *CBP60g* and *ICS2* in leaves of WT, *atnap*, *sag202* and *ics1* null mutants at different senescence stages.** Relative expression levels were calculated and normalized with respect to *Actin2* (*ACT2*) transcripts. Error bar indicate SD of three biological repeats. Significant (*P*<0.05) differences between means are indicated by different letters using Tukey’s HSD test.

**Supplementary Table S1 Primers used in this research**

| **Name** | **Sequence** (5’🡪3’) | **Engineered Restriction Enzyme Sites** | **Purpose** |
| --- | --- | --- | --- |
| G3830 | CTGCAGTAGAGAATGTCAGTTATATGATTGGC | the underlined section is an engineered PstI site | *P_SAG202_-GUS* |
| G3831 | CCATGGGGAATTGTTCTGGTGAGTTGTG | the underlined section is an engineered NcoI site |  |
| G3828 | CTGCAGATGGCAGGGAAGAGGTTATTTC | the underlined section is an engineered HindIII site | *SAG202* inducible lines |
| G3829 | GAGCTCTTAGAAAGGGTTTATATGATTTTG | the underlined section is an engineered PstI site |  |
| G4020 | AAGCTTACATGGCAGGGAAGAGGTTAT | the underlined section is an engineered HindIII site | *GAD-SAG202* |
| G3992 | CTCGAGCTTCCAATACTAACGTAGATGAGGAT | the underlined section is an engineered XhoI site |  |
| G3967 | GAATTCGCACGACAAGTTTTGAGAGGATG | the underlined section is an engineered EcoRI site | *P_SAG202_-LacZi* |
| G3918 | GTCGACGGAATTGTTCTGGTGAGTTGTG | the underlined section is an engineered SalI site |  |
| G3957 | GTCGACATACGATCGATCCGGTCCGT | the underlined section is an engineered SalI site |  |
| G3958 | GAATTCGGAAGATCGGAACCGTCCAT | the underlined section is an engineered EcoRI site |  |
| G4083 | CTCGAGACAACCTGGCAATATCCAAAG | the underlined section is an engineered XhoI site |  |
| G4108 | GAATTCGTCTCCCTATTTATGACGCCAT | the underlined section is an engineered EcoRI site |  |
| G4109 | GAATTCGATTATTCGCGTGGATCAGACTTCGG | the underlined section is an engineered EcoRI site |  |
| G3993 | GAATTCAAGATTTCGAACTAAAGC | the underlined section is an engineered EcoRI site | *P_ICS1_-LacZi* |
| G3994 | CTCGAGATAGGGGACTGATGTAGCAG | the underlined section is an engineered XhoI site |  |
| G4028 | GAATTCGGATTATCTGCAAGACTTC | the underlined section is an engineered EcoRI site |  |
| G4029 | GAATTCGGTGAGCCGTCTTAATC | the underlined section is an engineered EcoRI site |  |
| G4031 | GAATTCGAGCCTAAGTGGGTTTCC | the underlined section is an engineered EcoRI site |  |
| G4032 | CTCGAGGCAAAGAGTGGAGAGG | the underlined section is an engineered XhoI site |  |
| G4079 | GAATTCGTGCAAACCGCTTCCGTATCAAAC | the underlined section is an engineered EcoRI site |  |
| G4080 | CTCGAGGGGTGTTGTATGCTAGACGACTCTTC | the underlined section is an engineered XhoI site |  |
| G3149 | CGTAAAGCATCAACGAAACG |  | *AtNAP* qPCR |
| G3835 | TGGAAGTTTCATCGACGTCAT |  |  |
| G3875 | CCTCAACCAGCCCTACGTTA |  | *SAG202* qPCR |
| G3876 | TAGTGGCTCGCAGCATATTG |  |  |
| G3877 | GTGACATCATGGTTCTCTGTTACC |  | *ICS1* qPCR |
| G3878 | AGGCCTGCCCTAGTTACAACC |  |  |
| G3826 | AGCAATCGATTGGCTTCAT |  | *ICS2* qPCR |
| G3827 | TCCATAGGCACGAATCAGAG |  |  |
| G4061 | AGGTCCTTACAGTGCCCGCAAG |  | *CBP60g* qPCR |
| G4062 | CAGGGTGGACCGTTGAGCTTG |  |  |
| G3053 | AGTGGTCGTACAACCGGTATTGT |  | *Actin2* qPCR |
| G3054 | GATGGCATGAGGAAGAGAGAAAC |  |  |
